# Inhibition of mesenchymal drift as a strategy for rejuvenation

**DOI:** 10.3389/fphar.2025.1715559

**Published:** 2025-11-13

**Authors:** Lucas Paulo de Lima Camillo

**Affiliations:** 1 School of Clinical Medicine, University of Cambridge, Cambridge, United Kingdom; 2 Shift Bioscience, Cambridge, United Kingdom

**Keywords:** aging, rejuvenation, EMT, reprogramming, OSKM

## Abstract

Mesenchymal drift (MD), the progressive acquisition of mesenchymal traits by epithelial and endothelial cells, has emerged as a unifying mechanism of aging. Transcriptomic analyses across human tissues reveal that mesenchymal programs intensify with age and predict morbidity and mortality. By eroding lineage identity and promoting fibrosis, MD disrupts organ integrity in the lung, liver, kidney, heart, and brain. Mechanistically, it converges with epigenetic erosion, chronic inflammation, and extracellular matrix stiffening to establish self-reinforcing loops of dysfunction. Interventions that restore cellular identity can suppress MD: transient reprogramming resets epigenetic age and reduces fibrotic signatures without loss of identity, while chemical cocktails achieve similar rejuvenation effects with enhanced translational potential. Together, these findings establish MD as a tentative hallmark of aging and suggest that its inhibition could represent a strategy for cellular and tissue rejuvenation.

## 1 Introduction

Cellular identity gradually erodes with age. Mesenchymal drift (MD) describes a prominent trajectory within this erosion: the progressive adoption of mesenchymal features by cells that should maintain epithelial, endothelial, or other specialized programs. Across tissues, MD signatures tend to increase with age and associate with morbidity and mortality, and they appear in diseases marked by fibrosis and impaired repair. Mechanistically, MD aligns with sustained TGF- β signaling, chronic inflammation, and matrix stiffening, forming feedback loops that erode lineage fidelity. The sections that follow synthesize current evidence and outline genetic and pharmacological strategies that may suppress MD while preserving cellular identity.

## 2 Mesenchymal drift in aging: definition and evidence

MD describes the progressive adoption of mesenchymal features, such as motility, extracellular matrix deposition, and loss of adhesion, by non-mesenchymal cells. In a pan-tissue analysis of 42 human tissues from 948 donors aged 20–79 years, Belmonte and colleagues reported that mesenchymal gene programs consistently intensified with age, with correlation coefficients exceeding 0.3 across nearly all tissues after controlling for demographic, technical, and compositional confounders (Lu et al., 2025). These findings support MD as a widespread molecular signature of aging rather than a tissue-specific phenomenon (Gorelov and Hochedlinger, 2024).

Typical triggers include TGF- β /SMAD signaling, chronic inflammatory cytokines (e.g., IL-1 β , IL-6/STAT3), and matrix stiffening with YAP/TAZ activation. A core marker set comprises upregulation of mesenchymal genes (e.g., *VIM*, *FN1*, *COL1A1/3A1*, context-dependent *ACTA2*, *SNAI*/*ZEB* families) alongside loss of lineage markers (e.g., EPCAM, E-cadherin/*CDH1*, claudins; in endothelium, PECAM1/CD31, VE-cadherin/CDH5). Functional readouts include weakened barrier function, increased contractility/motility, and extracellular matrix deposition. Unlike classical epithelial/endothelial-to-mesenchymal transitions (EMT/EndoMT), which are tightly regulated, switch-like programs in development and cancer, MD is low-grade, mosaic within single cells and populations, and unfolds over months to years; critically, it appears partially reversible by TGF- β blockade, matrix softening, or pro-MET interventions.

Mechanistically, MD reflects dysregulated plasticity that erodes lineage fidelity and compromises tissue homeostasis. Hallmark features include cytoskeletal remodeling, heightened contractility, and breakdown of cell-cell junctions, which together disrupt organ architecture and function (Khalil and Nieto, 2024). Inter-tissue comparisons further show that gene expression profiles converge with age, reducing tissue specificity and amplifying systemic MD signatures (Izgi et al., 2022). Conceptually, this distinguishes MD (chronic, partial, multi-lineage, aging-associated) from classical EMT/EndoMT (acute, switch-like, developmental/cancer contexts).

Clinically, MD offers a unifying framework linking chronological aging to disease across multiple organ systems. In idiopathic pulmonary fibrosis (IPF), single-cell analyses reveal both compositional shifts and within-cell reprogramming of epithelial and endothelial populations. In a complementary bronchoalveolar lavage (BAL) gene-expression cohort of IPF patients (n = 132), stratification by MD-gene levels yielded median survival times of 2,498, 1,027/604, and 59 days across low, middle, and high MD groups, respectively (Lu et al., 2025). In the liver, MD rises progressively along the spectrum from metabolic dysfunction-associated steatotic liver disease (MASLD) to cirrhosis, with hepatocytes and stellate cells showing graded activation (Man-Chung Li et al., 2020). In chronic kidney disease, proximal and distal tubules and podocytes acquire mesenchymal features, while in heart failure, MD signatures reliably distinguish failing from non-failing myocardium. Plasma proteomic analysis of aging cohorts shows enrichment for EMT and TGF- β pathways, with proteins such as GDF15, pleiotrophin, and eotaxin-1 displaying the strongest associations with high MD states (Lu et al., 2025). In the UK Biobank, mortality-associated proteins exhibit strong EMT-related pathway enrichment, further implicating MD-linked biology in organismal decline. Finally, aging hematopoietic niches demonstrate pro-fibrotic remodeling and myeloid skewing in stromal cells, highlighting the contribution of bone marrow environments to systemic MD propagation (Ho et al., 2019). Together, these data support MD as an emerging candidate aging mechanism and a clinically actionable biomarker for prognosis and therapeutic targeting.

## 3 Partial reprogramming and MD suppression

Genetic partial reprogramming provides evidence that MD may be reversible and mechanistically linked to epigenetic aging. Lu et al. reported that suppression of the EMT regulator ZEB1 in human fibroblasts reduced DNA methylation-based age signatures, consistent with a link between MD and epigenetic dysregulation (Lu et al., 2025; Yang et al., 2023a). Importantly, transient expression of the Yamanaka factors (OSKM) for 1 week suppressed MD before activation of pluripotency markers such as NANOG, restoring cellular function without erasing lineage identity. This intermediate state was associated with early mesenchymal-to-epithelial transition (MET), reduced β -galactosidase activity by 40%–60%, and rejuvenated transcriptional profiles within 7 days (Lapasset et al., 2011; Takahashi and Yamanaka, 2006). These observations suggest a therapeutic window in which reprogramming reverses aging signatures without inducing dedifferentiation, broadly consistent with the literature (Gill et al., 2022).

Safer genetic variants indicate that MD suppression can be decoupled from tumorigenic risk. Removal of the oncogene MYC to form OSK (OCT4, SOX2, KLF4) preserved MD suppression while lowering oncogenic potential, while an engineered OCT4 YR mutant unable to dimerize with SOX2 retained the positive MD reversal without activating NANOG or inducing pluripotency (Lu et al., 2025; Lu et al., 2020). An alternative factor to OSKM dubbed SB000 has also been shown to rejuvenate human fibroblasts and keratinocytes in a multiomic analysis (de Lima Camillo et al., 2025). Interestingly, the main hypermethylated pathway after 6 weeks of induction (inhibition of expression) was EMT. These refinements suggest that not all reprogramming factors are required to achieve MD reversal, and that functional rejuvenation may be achievable without crossing into pluripotency.

*In vivo* studies indicate that partial reprogramming can reduce MD and restore organ function in aged animals. Cyclic OSKM induction in aged mice lowered MD scores in kidney and liver by 25%–40% after 2–4 weeks, improving tissue homeostasis without teratoma formation (Ocampo et al., 2016; Browder et al., 2022). Functional rescue has been observed in disease models: OSK delivery restored vision in about half of glaucomatous mice aged 12–18 months (Lu et al., 2020), while transient OSKM induction in cutaneous wounds reduced scar formation by 30%–50%, promoting regenerative healing (Doeser et al., 2018). In progeroid ERCC1-deficient mouse cells, initiation-phase reprogramming reduced DNA damage and restored epigenetic age, underscoring the potential benefits of controlled genetic reprogramming (Treat Paine et al., 2024).

In practice, *in vivo* reprogramming effects generally arise from short pulses applied over weeks (e.g., multi-week 2-day-on/5-day-off OSKM cycles), with primary endpoints including organ function, histology, and molecular aging clocks. Known liabilities include dedifferentiation and tumorigenic risk with c-MYC; OSK or engineered factor variants mitigate but do not eliminate this risk. Careful temporal control and biomarker monitoring remain essential for translation.

Taken together, current evidence supports genetic partial reprogramming as a promising strategy to suppress MD and restore more youthful epigenetic and functional states. By exploiting temporal windows and refining factor combinations, this approach may achieve rejuvenation while minimizing the risks of dedifferentiation and tumorigenesis, making it a plausible cornerstone for translational interventions in aging biology.

## 4 Pharmacological therapeutic approaches

Pharmacological interventions offer a tractable route to suppress MD by targeting the signaling, mechanical, and systemic loops that sustain it. Aging tissues accumulate extracellular matrix (ECM) modifications such as non-enzymatic glycation and collagen cross-linking that increase stiffness and promote TGF- β and inflammatory signaling, thereby reinforcing MD (Fedintsev and Moskalev, 2020; Pardo and Pardo, 2021; Rice et al., 2017). These processes drive maladaptive transitions, including EndoMT, which weakens barrier function through loss of VE-cadherin, and macrophage-to-myofibroblast conversion, which accelerates fibrosis *via* TGF- β /SMAD3 activation (Trogisch et al., 2024; Haider et al., 2019; Jia, 2025). These changes create a feed-forward cycle in which local injury and systemic inflammation amplify MD across multiple cell types.

Targeting TGF- β signaling remains a direct strategy for MD inhibition. Small-molecule receptor inhibitors, SMAD3 antagonists, and ligand traps reduce ZEB and SNAI expression while restoring epithelial markers such as EPCAM and claudins (Massagué, 2012; Deng et al., 2024). Interestingly, RepSox, a TGF- β receptor inhibitor, has a dual identity: it both suppresses MD by blocking profibrotic signaling and serves as a substitute for SOX2 in chemical reprogramming cocktails (Ichida et al., 2009). This highlights a mechanistic bridge between rejuvenation strategies and MD suppression, underscoring how reprogramming interventions may derive part of their efficacy from direct modulation of TGF- β pathways. Beyond RepSox, ALK5/ALK2 inhibitors can partially phenocopy the benefits of OSKM initiation-phase reprogramming in ERCC1-deficient progeroid mice, restoring DNA repair and resetting the epigenetic clock (Treat Paine et al., 2024). These findings suggest that pharmacological blockade of TGF- β receptors may mimic core aspects of genetic partial reprogramming.

Beyond monotherapies, combination regimens that recalibrate systemic pro-youth signals are gaining momentum. Notably, in 25-month-old frail mice, daily subcutaneous oxytocin combined with an ALK5 inhibitor (OT + A5i) extended remaining lifespan by approximately 73% from treatment onset and increased overall median lifespan by about 14% in males, with concomitant healthspan improvements (Kato et al., 2025). As ALK5 blockade directly attenuates TGF- β signaling, a key driver of MD, these results suggest that pharmacologic MD suppression may extend health- and lifespan, at least in males.

Complementary strategies strengthen these effects through parallel mechanisms. Inflammation-targeting drugs include IKK inhibitors that block NF- κ B-driven IL-6/IL-8 signaling, IL-1 β antagonists that blunt upstream cytokine cascades, and STAT3 inhibitors that interrupt IL-6-dependent feedback. Mechanical interventions act in parallel by reducing cytoskeletal contractility and ECM stiffening. For example, ROCK inhibitors restore E-cadherin expression and reduce stress-fiber formation (Rath and Olson, 2012), while collagen cross-link breakers such as alagebrium decrease hydroxylysyl-pyridinoline cross-links, although clinical results remain mixed. Inhibitors of lysyl oxidase, including simtuzumab, prevent new cross-linking but have not demonstrated efficacy in late-stage fibrosis trials, underscoring the challenge of targeting advanced lesions. Practical liabilities for TGF- β pathway inhibitors include potential impairment of wound healing and immunomodulatory effects; biomarker-led patient selection and limited-duration dosing may help mitigate risks.

Cell-type specific approaches further refine therapeutic potential. Preventing EndoMT maintains endothelial integrity, while blocking macrophage-to-myofibroblast conversion limits fibrosis at its source. Recent studies also identified matrix-producing neutrophils in the skin, regulated by TGF- β , that contribute to barrier remodeling and may represent novel pharmacological targets (Vicanolo et al., 2025). Combining TGF- β inhibitors such as RepSox or ALK-blockers with epigenetic modulators offers temporal MD suppression without inducing dedifferentiation (Yang et al., 2023b; Mitchell et al., 2024; Oliviero, 2019).

In sum, pharmacological approaches to MD inhibition extend beyond classical antifibrotic strategies: they converge with reprogramming mechanisms, revealing shared pathways between aging reversal and fibrosis suppression. By leveraging agents like RepSox and ALK inhibitors, it is possible to mimic key aspects of partial reprogramming pharmacologically, opening the door to safer, more readily translatable interventions for systemic rejuvenation.

## 5 Conclusion

MD provides a conceptual and mechanistic bridge between aging and age-related disease, positioning loss of cellular identity as a root cause of organismal decline. Genetic partial reprogramming demonstrates that MD is reversible, with controlled OSK/OSKM induction restoring youthful function in multiple organs, while chemical cocktails and pharmacological inhibitors such as RepSox or ALK5/ALK2 blockers achieve similar effects with enhanced safety. Together, these strategies reveal that MD suppression may reset epigenetic age, reduce fibrosis, and improve organ function across systems. Future progress will depend on refining temporal control, minimizing oncogenic risk, and integrating pharmacological and reprogramming approaches into clinically viable interventions.
